# Association between gut microbiota and hepatocellular carcinoma from 2011 to 2022: Bibliometric analysis and global trends

**DOI:** 10.3389/fonc.2023.1120515

**Published:** 2023-03-30

**Authors:** Zhitao Chen, Chenchen Ding, Yangjun Gu, Yahui He, Bing Chen, Shusen Zheng, Qiyong Li

**Affiliations:** ^1^ Department of Hepatobiliary Surgery, Shulan (Hangzhou) Hospital Affiliated to Zhejiang Shuren University Shulan International Medical College, Hangzhou, China; ^2^ Affiliated Mental Health Centre & Hangzhou Seventh People’s Hospital, Zhejiang University School of Medicine, Hangzhou, Zhejiang, China; ^3^ School of Medicine, Zhejiang Chinese Medical University, Zhejiang Shuren College, Hangzhou, China; ^4^ Jinan Microecological Biomedicine Shandong Laboratory, Jinan, China

**Keywords:** gut microbiota, hepatocellular carcinoma, bibliometric analysis, publications, average citation

## Abstract

**Background:**

Hepatocellular carcinoma (HCC) is a primary malignant tumor responsible for approximately 90% of all liver cancers in humans, making it one of the leading public health problems worldwide. The gut microbiota is a complex microbial ecosystem that can influence tumor formation, metastasis, and resistance to treatment. Therefore, understanding the potential mechanisms of gut microbiota pathogenesis is critical for the prevention and treatment of HCC.

**Materials and methods:**

A search was conducted in the Web of Science Core Collection (WoSCC) database for English literature studies on the relationship between gut microbiota and HCC from 2011 to 2022. Bibliometric analysis tools such as VOSviewer, CiteSpace, and R Studio were used to analyze global trends and research hotspots in this field.

**Results:**

A total of 739 eligible publications, comprising of 383 articles and 356 reviews, were analyzed. Over the past 11 years, there has been a rapid increase in the annual number of publications and average citation levels, especially in the last five years. The majority of published articles on this topic originated from China (n=257, 34.78%), followed by the United States of America (n=203, 27.47%), and Italy (n=85, 11.50%). American scholars demonstrated high productivity, prominence, and academic environment influence in the research of this subject. Furthermore, the University of California, San Diego published the most papers (n=24) and had the highest average citation value (value=152.17) in the study of the relationship between gut microbiota and HCC. Schnabl B from the USA and Ohtani N from Japan were the authors with the highest number of publications and average citation value, respectively.

**Conclusion:**

In recent years, research on the gut microbiota’s role in HCC has made rapid progress. Through a review of published literature, it has been found that the gut microbiota is crucial in the pathogenesis of HCC and in oncotherapy.

## Introduction

Liver cancer is a common digestive malignant cancer worldwide (1). According to accumulated data, every year approximately 841,000 new cases of liver cancer are diagnosed and nearly 1 million people die from diseases related to liver cancer (1, 2). Hepatocellular carcinoma (HCC) with high fatality rate is the most prevalent clinicopathological type of liver cancer (3). Most patients with HCC in China are at intermediate-advanced stages when diagnosed, in which effective treatment strategies are limited and with negative results, with an extremely poor prognosis (4). Therefore, uncovering the potential biological mechanisms of hepatocellular carcinogenesis may be useful for cancer comprehensive therapy and is urgently required.

In recent years, several studies have focused on the connection between intestinal flora and diseases (5). Regulating the intestinal flora and improving gut barrier function has gradually become a novel and exciting method for the preventing and curing some diseases (6). As the hepatic portal vein and bile secretion systems directly links the liver to the gut, this association might be relevant to find specific associations between liver diseases and intestinal flora. Hepatocytes are unremittingly exposed to a wide range of intestinal flora and microbe-associated molecular patterns that influence the stability of hepatic microenvironments, thereby driving the initiation and progression of liver disease, including nonalcoholic fatty liver disease (NAFLD) (7). It has been previously established that intestinal microbiota depletion by administration of broad-spectrum antibiotics suppress experimental nonalcoholic steatohepatitis (NASH), which is one of common causes of HCC in developed countries (8, 9). In contrast, the regulation of distinct intestinal flora can influence anti-tumor immunotherapy responses, including HCC (9). Also, the dynamic gut microbiota variation characteristics may be exploited as a potent biomarker to early predictions immunotherapy outcomes in HCC, which is critical for HCC surveillance and cancer immunotherapy decision-making (9). So, it is crucial to explore a relationship between gut microbiota and hepatocarcinogenesis that is beneficial to understand both the molecular mechanisms of tumor formation and tumor treatment.

Currently, numerous scientific methods are used to systematically understand the current status of an academic field, of which bibliometric analysis using mathematical and statistical methods has become the most prevalent tool to acquire overall knowledge structure and research priorities in an academic domain (10, 11). In recent years, with the deepening of intestinal flora in tumorigenesis and cancer treatment, studies have revealed that the unbalance of intestinal flora is closely correlated to HCC (5, 9). Although the research on the intestinal flora were previously explored in HCC based on numerous experiments, no systematic analysis of global research trends in this specific academic fields. Bibliometrics can not only describe the current research status but also assess research prospects and predict new tendencies in current academic research, which cannot be achieved using other methods including meta-analyses, conventional reviews or experimental studies. Hence, in the present study, we first used bibliometric methods to analyze the current status and future research directions of the intestinal flora research in HCC domain.

## Materials and methods

### Ethics statement

This study was based on published papers from January 01, 2011 to October 26, 2022 available in the public domain and did not involve human or animal studies or experiments. So, this research did not require ethical approval.

### Data source and retrieval strategies

**Figure 1:**
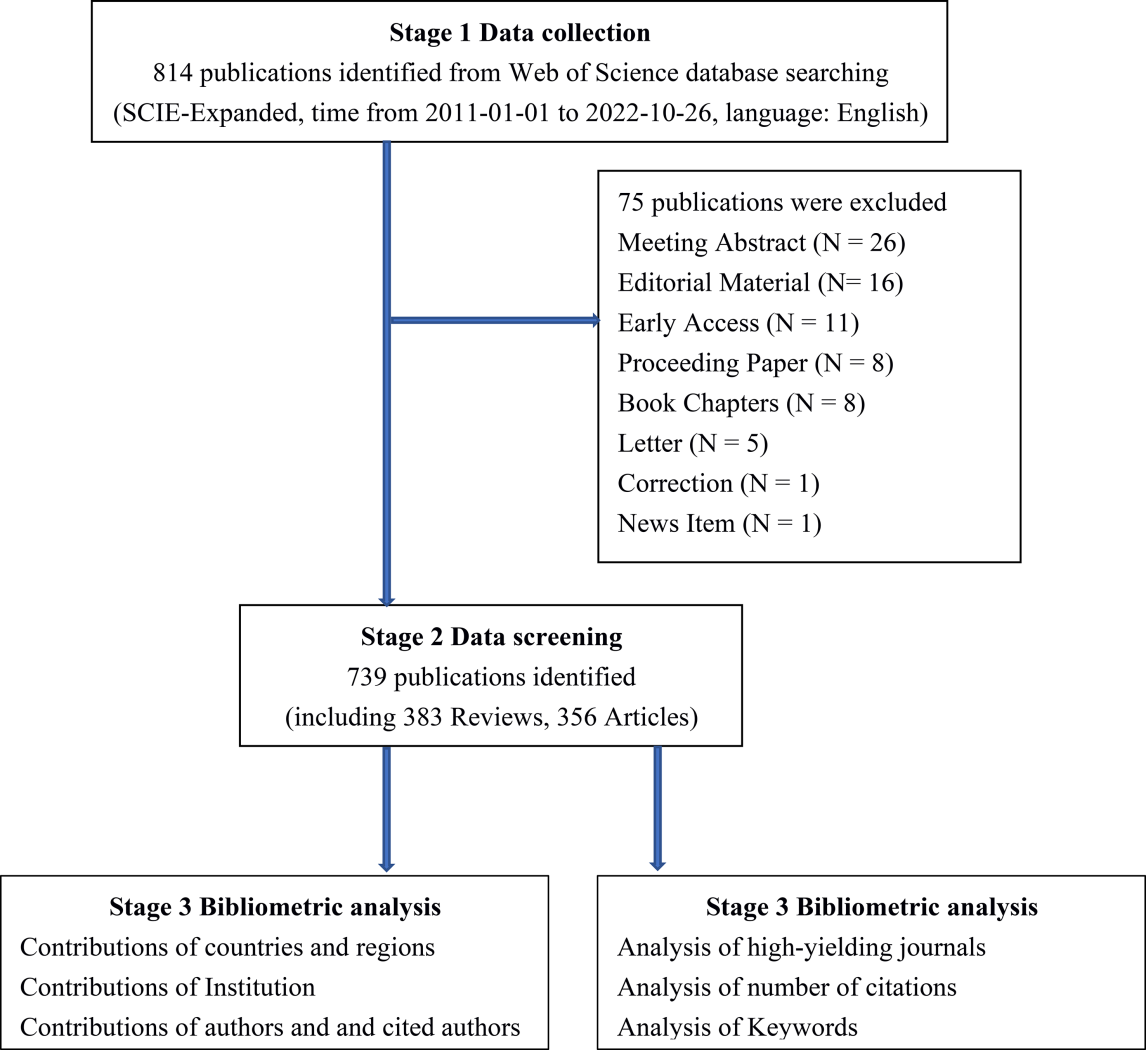
Detailed flowchart of search.

We conducted a comprehensive literature search for all included studies of gut microbiota in HCC in Web of Science Core Collection (WoSCC, https://www.webofscience.com/) database. The search was independently searched and extracted by coauthors (ZC and CD) and excluded those that did not fit according to the following inclusion and exclusion criteria: (1) The publications were from the WoSCC Science Citation Index Expanded (SCI-E) databases. (2) Language restriction for the publication is in English. (3) The types of publications are restricted to original articles and reviews. (4) Excluding other types of publications, including meeting abstract, editorial material, early access, proceeding paper, book chapters, letter, correction, news item. (5) The retrieval time range is from January 01, 2011 to October 26, 2022. (6) The subjects of the publication were HCC patients, HCC animal models, and HCC cellular models, and the studies must also assess the correlation between subjects and gut microbiota. The detailed WoSCC database search strategy is summarized in **Table 1**. Finally, 739 eligible publications, including 383 articles and 356 reviews, were included in the subsequent analysis (**Figure 1**).

**Table 1:** The search strategy of Web of Science Core Collection (WoSCC).

Research database	Web of Science Core Collection
Citation indexes	Science Citation Index Expanded
Query formulation	(((TS=(gut OR intestin* OR gastrointestin*)) AND TS=(microbio* OR microflora OR flora OR bacteri* OR dysbiosis OR microecology OR 16Sr* OR metagenome)) OR TS=(prebiotic* OR probiotic* OR synbiotic*)) AND TS=(Hepatocellular carcinoma OR Hepatocellular carcinomas OR HCC)
Language	English
type of aricles	Articles and Reviews
Searching period	January 01, 2011 to October 26, 2022
Data collection	export with full records and cite reference in plain text format
Sample size	739 publications including 383 articles and 356 reviews.

* wildcard.

### Data analyses and visualization

The WoSCC intrinsic toolkits were used to analyze general characteristics of eligible literatures, including the list of Web of Science subject categories, the number of annual publications, citations and the h-index. Data analysis and visualization, the mainstream bibliometric analysis tools include CiteSpace (12), VOSviewer (11), R software, SCI2, and HistCite. There is no consensus as to which approach of bibliometric analysis is the best. Therefore, the respective properties and advantages for these tools were combined together, we used VOSviewer, R software and CiteSpace at the same time for next analysis. VOSviewer (11) (Leiden University, Leiden, The Netherlands) is a powerful software for bibliometric network construction and visualization based on publications, countries, authors, journals and keywords. And the burst keywords for forecasting the possible hotspots was explored *via* CiteSpace (10) (Drexel University, Philadelphia, PA, USA). Meanwhile, the R package bibliometrix was used to output the collaboration map of countries in gut microbiota and HCC.

## Results

### Annual tendencies and annual citations

**Figure 2:**
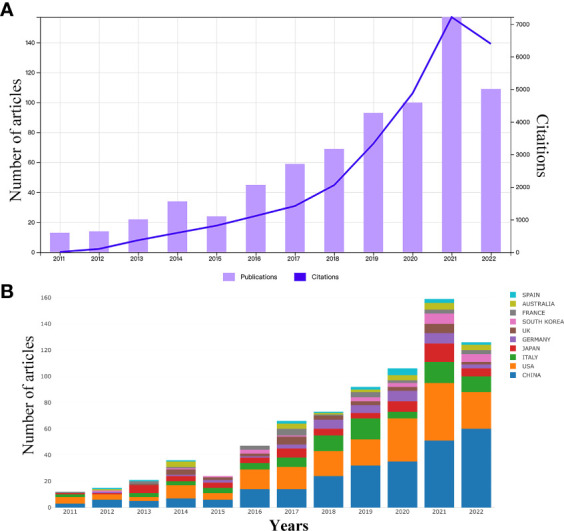
**(A)** The number of annual research publications and growth trends on the links of gut microbiota and HCC from 2011 to 2022. **(B)** Trends in the proportion of annual research publications in different countries.

According to the search strategy (**Table 1**), a total of 814 eligible publications were collected from the time of January 01, 2011 to October 26, 2022 in WoSCC database. Subsequently, 75 literatures were extracted, including meeting abstract (N = 26), editorial material (N= 16), early access (N = 11), proceeding paper (N = 8), book chapters (N = 8), letter (N = 5), correction (N = 1), news item (N = 1). Finally, 739 eligible publications, including 383 articles and 356 reviews, were included in the subsequent analysis (**Figure 1**). The research on gut microbiota in HCC has continuously and steadily increased over the past 10 years, with annual growth rate 22.67% (**Figure 2A**). The number of published papers and the corresponding citations have exponentially increased year by year, and the number of both in 2021 was the largest (157 publications and 7215 citations), indicating that gut microbiota has gradually gained considerable attention in the field of HCC research (**Figure 2A**). In the field of gut microbiota in HCC research, the China and United States of America (USA) published the most research results (**Figure 2B**). As of October 26, 2022, the 739 publications were cited 28,227 times which is an average 38.2 citations per publication. Meanwhile, we found that these publications in “gut microbiota and HCC” domains cited 46,843 expanded literature from 4,640 different journals.

### Distribution of countries/regions

**Figure 3:**
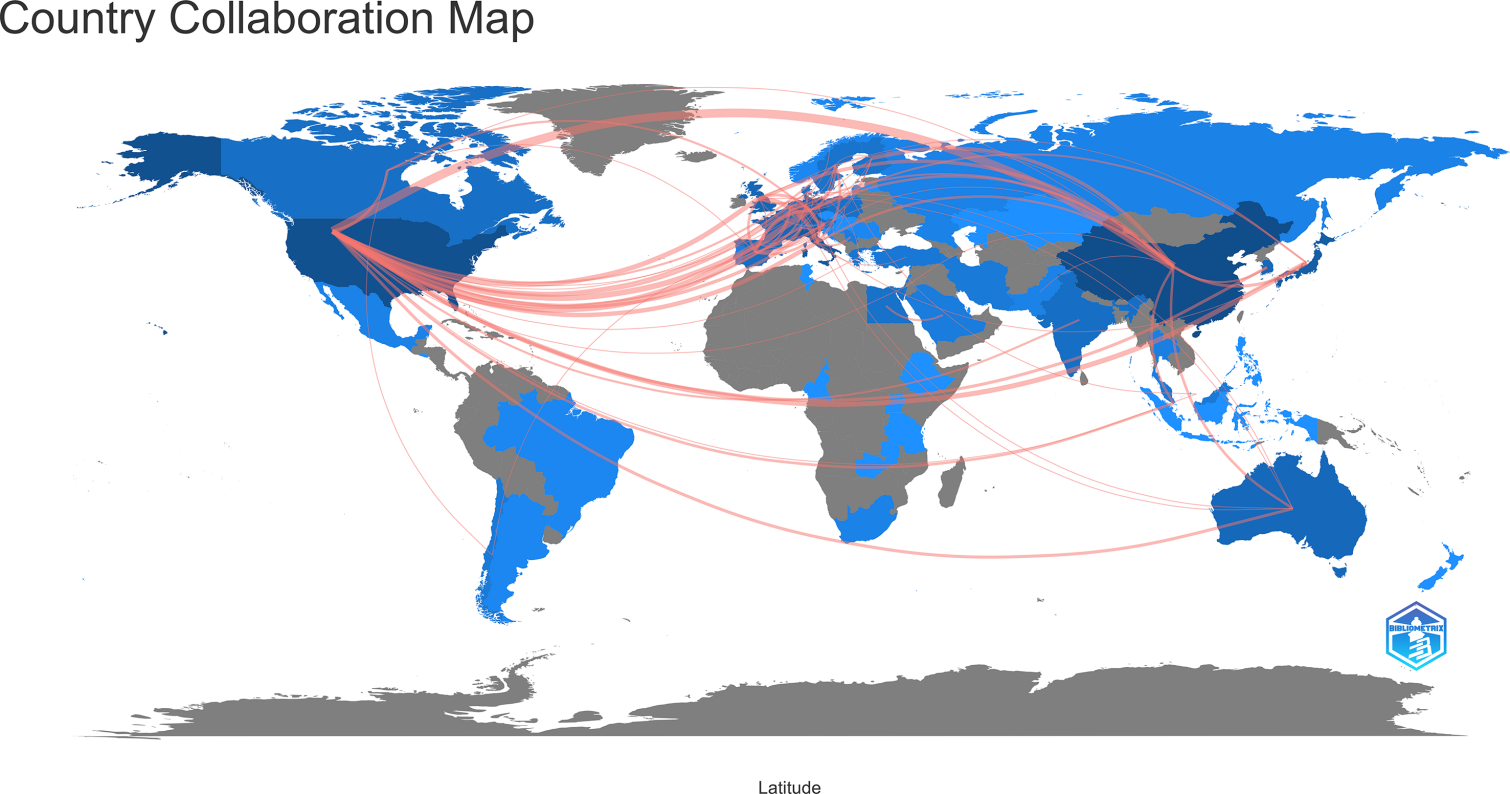
The network map of collaboration relations between countries generated with R software.

**Figure 4:**
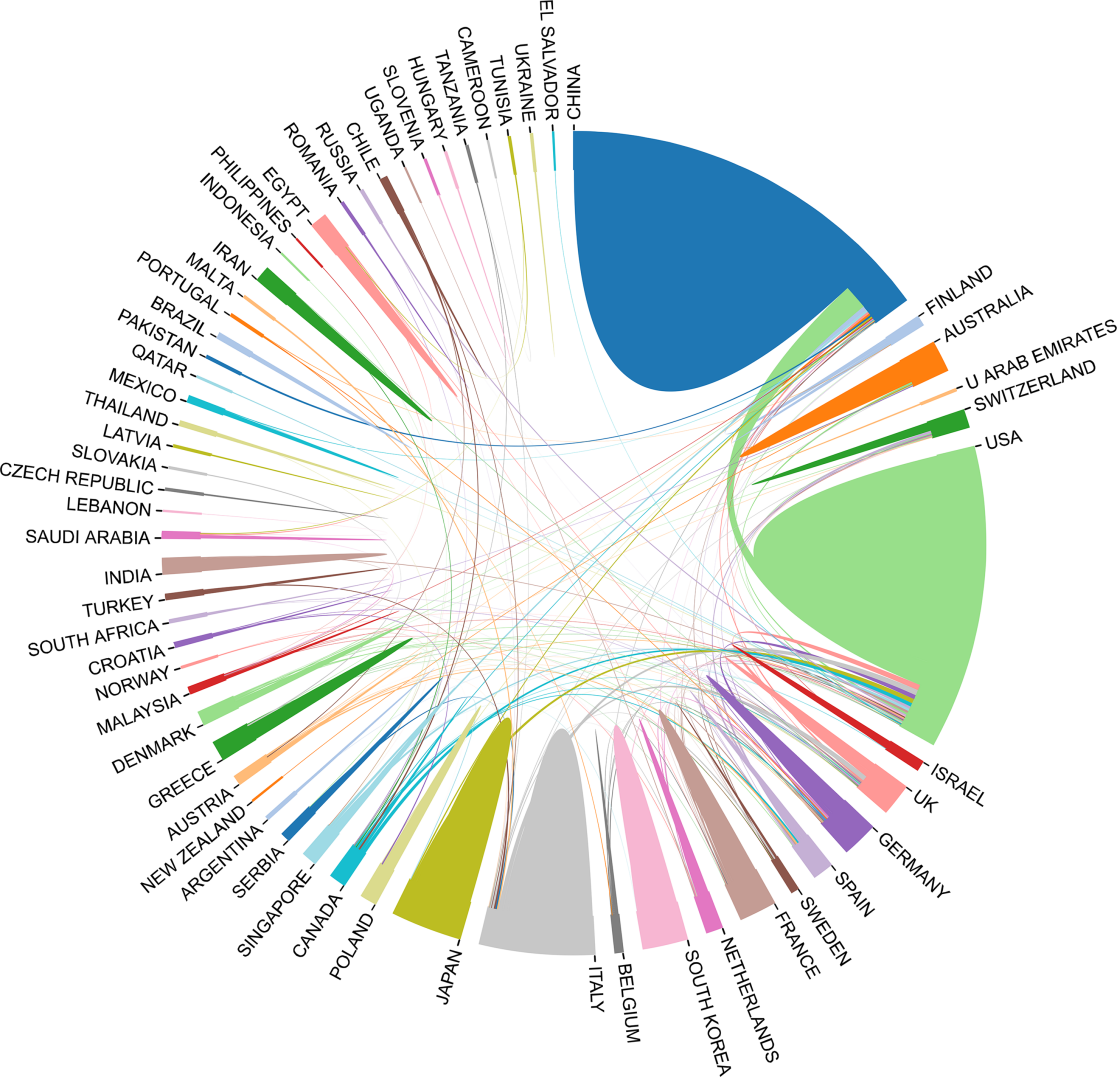
Cooperative relationships between different countries on the connection of gut microbiota and HCC generated with R software.

**Figure 5:**
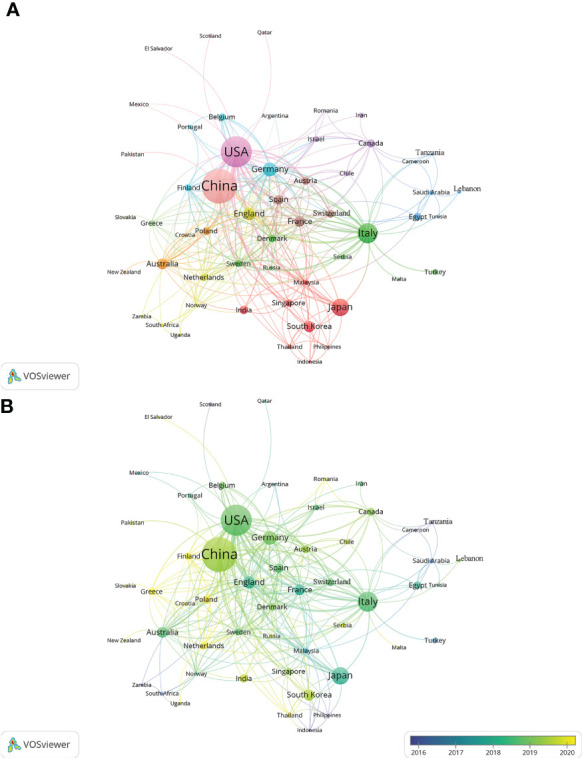
Cooperative relationships between different countries on the connection of gut microbiota and HCC generated with VOSviewer. **(A)** The network of different countries visualization. Each node represents one country. Circle size is based on the number of publications. Connecting lines represent collaboration between countries. Different colors represent different items. **(B)** Present the distribution of countries according to the average time of occurrence. Green and blue circles indicate more past publications, and yellow circles indicate more recent publications.

In the past 11 years, a total of 1,155 research institutions from 64 countries have published gut microbiota and HCC related publications. We found that these articles were from 334 journals and 4296 authors. Information from the published articles was used to create a world map illustrating the cooperative relationship of the research topic in different countries (**Figure 3**). The countries were ranked in relation to number of published articles, and only the top ten countries are presented. According to the **Table 2**, we found that the majority of published articles regarding this topic have their origin in the China (n = 257, 34.78%), followed by United States of America (n = 203, 27.47%), and Italy (n = 85, 11.50%). Looking at number of published papers, these three countries have made indelible contributions in the gut microbiota and HCC field. Based on the number of publications by researcher, one thing worth noting is that USA has the second number of publications in this field and the highest average citation score, indicating that research institutions in USA is important research forces in this field. Although China has the highest number of publications, but its average citation score is 28.96. Meanwhile, **Figures 4**, **5** present the collaboration map of countries in gut microbiota and HCC. The result shows that China largely cooperated with USA, England, Australia, Italy, and Japan. The result also reveals that scientific research respects no geographical boundaries, so international collaboration in conducting studies in the field will be important.

**Table 2:** Top 10 productive countries related to gut microbiome in HCC related research.

Rank	Country	Documents (n)	Percentage (n/739)	Citations	Average citations
1	China	257	34.78%	7442	28.96
2	USA	203	27.47%	12166	59.93
3	Italy	85	11.50%	3925	46.18
4	Japan	65	8.80%	3474	53.45
5	Germany	40	5.41%	1794	44.85
6	England	32	4.33%	1449	45.28
7	South Korea	27	3.65%	1151	42.63
8	Australia	25	3.38%	1103	44.12
9	France	25	3.38%	751	30.04
10	Spain	18	2.44%	1379	76.61

HCC, Hepatocellular carcinoma.

### Distribution of institutions

**Figure 6:**
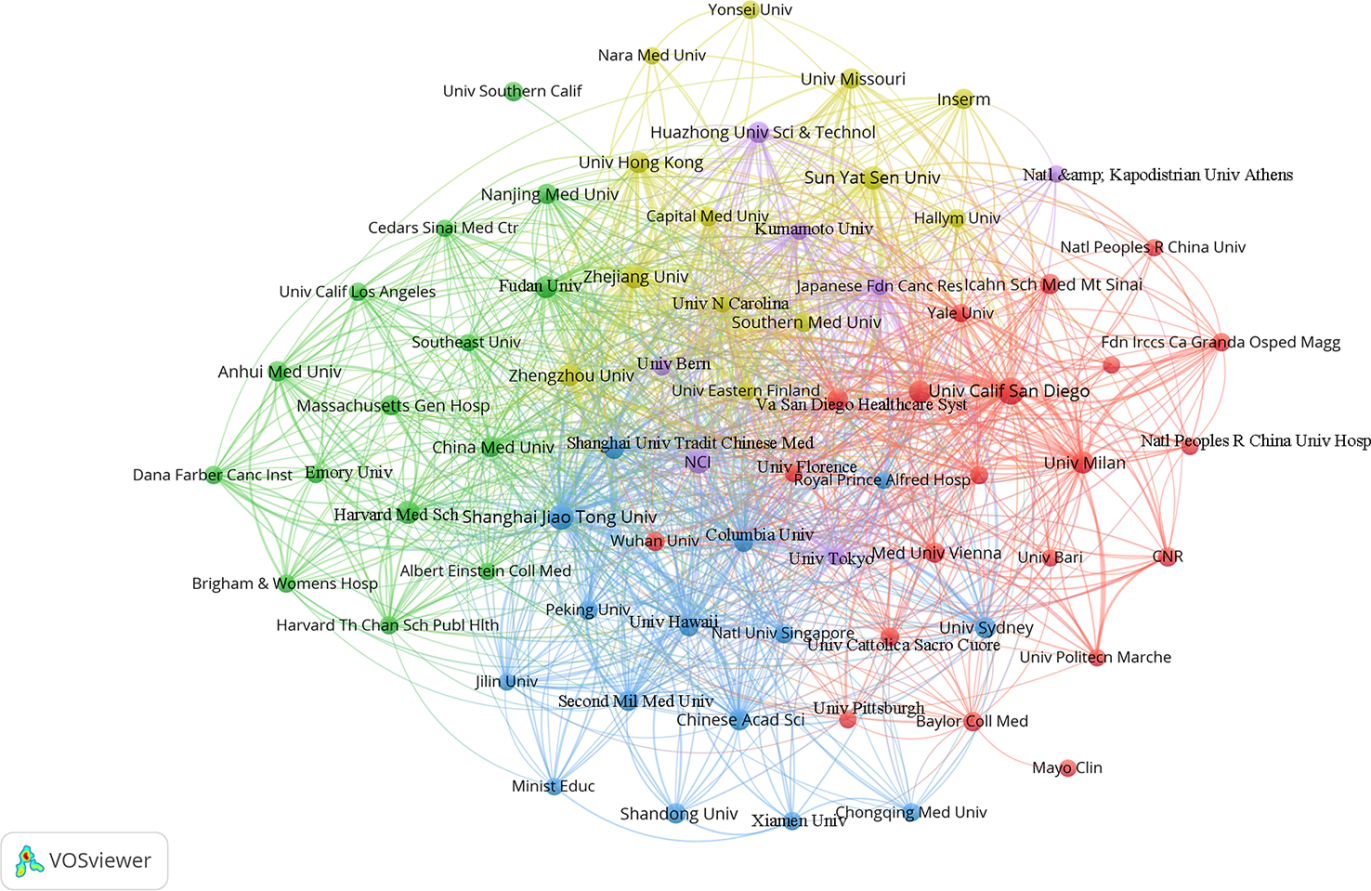
The co-authorship network visualization map of institutions. Each node represents one country. Circle size is based on the number of publications. Connecting lines represent collaboration between institutions, and the same color of node represent the same cluster.

VOSviewer was used to analyze and visualize the 1,155 research institutions that contributed to the field. Publications (the minimum number of documents used by an organization was defined as more than 5) were identified in the 71 institutions and visualized using VOSviewer (**Figure 6**). **Table 3** shows the top ten contributing research institutions. There is a close cooperative relationship among these institutions. In the past 11 years, more than half of the top 10 research institutions from China (6/10), followed by the USA (3/10). The results indicating that institutions in China and USA have made outstanding contributions in gut microbiota and HCC field. Additionally, University of California, San Diego published the most papers (n = 24) and has the highest average citation value (value = 152.17), followed by Shanghai Jiaotong University with 19 publications and second average citation (value = 81.00). The low junction of institutions network indicates the lack of inter-agency collaboration and further emphasizes the importance of collaboration.

**Table 3:** Top 10 institutions ranked by the numbers of publications.

Rank	Organization	Country	Documents	Citations	Average citation	Total link strength
1	University of California, San Diego	USA	24	3652	152.17	5
2	Shanghai Jiaotong University	China	19	1539	81.00	6
3	Sun Yat-sen University	China	15	608	40.53	4
4	Harvard medicine school	USA	14	888	63.43	6
5	National Cancer Institute	USA	14	478	34.14	5
6	Zhejiang University	China	14	814	58.14	3
7	The Chinese University of Hong Kong	China	13	690	53.08	6
8	Fudan university	China	13	238	18.31	3
9	University of Milan	Italy	12	677	56.42	0
10	Zhengzhou University	China	12	375	31.25	6

### Contributions of authors and cited authors

**Figure 7:**
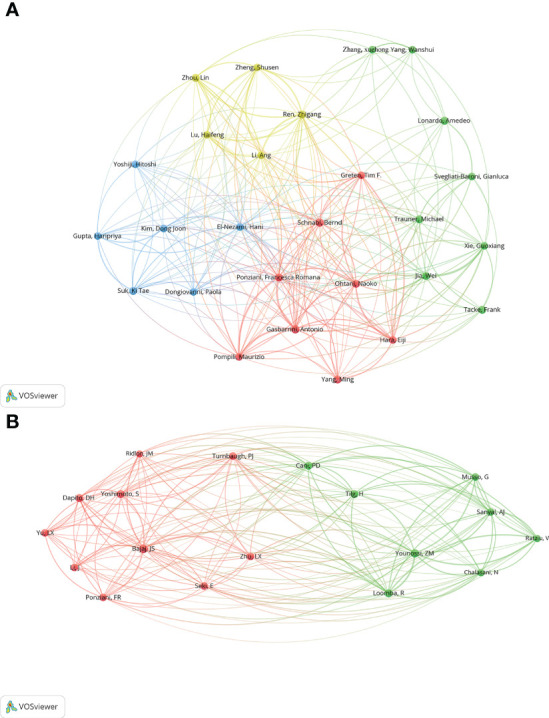
**(A)** Co-authorship network visualization map of authors. Circles represent the number of articles published. Connecting lines represent collaboration between authors. **(B)** Co-citation network visualization map of authors. The size of node and word reflects the co-occurrence frequencies, the link indicate the co-occurrence relationship, and the same color of node represent the same cluster.

Authors (n = 27) with a minimum productivity of 5 publications were visualized using VOSviewer software and showed in **Figure 7A**. Meanwhile, the collaboration relationship among these authors were displayed. The top 12 authors with the most active published articles also were listed (**Table 4**), including Schnabl B (9 articles, 725 citations), Gasbarrini A(8 articles, 360 citations), Jia W (7 articles, 879 citations), Ponziani FR (7 articles, 350 citations), Ren, ZG (7 articles, 484 citations), Tacke F (7 articles, 328 citations), Lonardo A (6 articles, 705 citations), Ohtani N (6 articles, 1600 citations), Suk KT (6 articles, 124 citations), Trauner M (6 articles, 561 citations), Yang M (6 articles, 55 citations), Zhou L (6 articles, 377 citations). Among these authors, high cited authors are from Asia (China and Japan). Ohtani N from Osaka Metropolitan University Graduate School of Medicine School of Medicine, Japan, is the author with the highest average citation, indicating that he is academically highly influential with outstanding contributions in the field. Jia W with second average citation from School of Chinese Medicine, Hong Kong Baptist University, Kowloon Tong, Hong Kong, also has extremely high academic influence in the field. The co-citation analysis of authors was performed by VOSviewer software (**Figure 7B**). A total of 18 authors were selected with more than 100 citations.

**Table 4:** The top 12 authors with the most active published articles.

Rank	Author	Country	Documents	Citations	Average citation
1	Schnabl B	USA	9	725	80.56
2	Gasbarrini A	Italy	8	360	45.00
3	Jia W	China	7	879	125.57
4	Ponziani FR	Italy	7	350	50.00
5	Ren, ZG	China	7	484	69.14
6	Tacke F	Germany	7	328	46.86
7	Lonardo A	Italy	6	705	117.5.
8	Ohtani N	Japan	6	1600	266.67
9	Suk KT	Korea	6	124	20.67
10	Trauner M	Austria	6	561	93.5
11	Yang M	China	6	55	9.17
12	Zhou L	China	6	377	62.83

### Analysis of high-yielding journals

**Figure 8:**
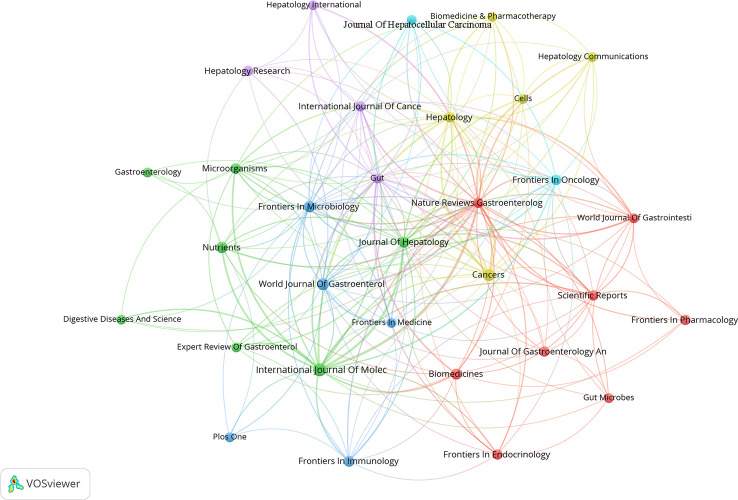
The co-authorship map of high-yielding journals. In the visualization map, one node represents a journal, and its size is proportional to the number of publications. Different colors represent different items. There are six items in the figure.

From the time of January 01, 2011 to October 26, 2022, 739 publications on research linked to gut microbiota and HCC were published in 334 journals, 31 journals of which contained at least 5 articles. The co-authorship analysis of journals was performed, and the network map was presented (**Figure 8**). We listed the top 10 journals with the most publications in this field (**Table 5**). Among the top ten journals, Nature reviews gastroenterology & hepatology has the highest impact factor (IF) with 73.082. Additionally, International journal of molecular sciences (2021 IF: 6.208), Cancers (2021 IF: 6.575), and World journal of gastroenterology (2021 IF: 5.374) are the journals with more than 20 publications each. Additionally, combined with the information provided by VOSviewer software and the Journal Citation Reports (JCR) assessment system, we also found that the top 10 journals are mainly concentrated in JCR of Q1(70%) and Q2 (30%), which indicates that these journals have a high degree of influence in the relevant domains. According to **Table 6**, Hepatology contributed the maximum quantity of publication citations with 4909. Journal of Hepatology was the second most cited journal, which had 2,858 citations. And it was followed by Gastroenterology, Nature, Gut.

**Table 5:** Top 10 journals with the highest number of articles.

Rank	Source	2022 IF	JCR	Publications	Citations
1	International journal of molecular sciences	6.208	Q1	37	1122
2	Cancers	6.575	Q1	22	156
3	World journal of gastroenterology	5.374	Q2	21	763
4	Frontiers in microbiology	6.064	Q1	17	493
5	Nutrients	6.706	Q1	14	283
6	Journal of hepatology	30.083	Q1	12	1170
7	Frontiers in immunology	8.786	Q1	11	316
8	Biomedicines	4.757	Q2	10	164
9	Frontiers in oncology	5.738	Q2	9	204
10	Nature reviews gastroenterology & hepatology	73.082	Q1	9	2193

IF, Impact Factor; JCR, Journal Citation Reports.

**Table 6:** Top 10 popular journals regarding the number of citations.

Rank	Source	2022 IF	JCR	Citations
1	Hepatology	17.298	Q1	4909
2	Journal of Hepatology	30.083	Q1	2858
3	Gastroenterology	33.883	Q1	2434
4	Nature	69.504	Q1	1687
5	Gut	31.793	Q1	1417
6	Plos One	3.752	Q2	1317
7	Science	63.714	Q1	1143
8	World Journal of Gastroenterology	5.374	Q2	1021
9	Proceedings of the National Academy of Sciences of the United States of America	12.779	Q1	988
10	Scientific Reports	4.996	Q2	856

IF, Impact Factor; JCR, Journal Citation Reports.

### Analysis of number of citations

The number of citations is a measure of impact of a publication in a scientific field. These 739 publications were counted and ranked by number of citations, and the top 10 publications are shown in **Table 7**. The most cited article was Jorge Henao-Mejia et al. (13) of Yale University published in 2012 with a citation number of 1557. The second cited article was also published in Nature in 2013. They believed that obesity can change the intestinal microbiota through senescence-associated secretory phenotype (SASP), which plays an important role in the development of HCC in mice (14). Additionally, the third cited article by Dianne H Dapito et al. (15) believed that gut microbiota has a profound influence on hepatocarcinogenesis in the chronically injured liver cells. Therefore, they also found that targeting the gut microbiota and TLR4 might ultimately improve primary or secondary prevention of HCC. These publications demonstrated that gut microbiota is a double-edged sword and plays a key role in tumorigenesis, progression and oncotherapy of HCC.

**Table 7:** The top 10 most cited articles in the connection of gut microbiota and HCC research from 2011 to 2022.

Article	Author	Years	Journals	2022 IF	DOI	Citations
Inflammasome-mediated dysbiosis regulates progression of NAFLD and obesity	Jorge Henao-Mejia	2012	Nature	69.504	10.1038/nature10809	1557
Obesity-induced gut microbial metabolite promotes liver cancer through senescence secretome	Shin Yoshimoto	2013	Nature	69.504	10.1038/nature12347	1230
Promotion of Hepatocellular Carcinoma by the Intestinal Microbiota and TLR4	Dianne H Dapito	2012	Cancer Cell	38.585	10.1016/j.ccr.2012.02.007	767
Bile acid–microbiota crosstalk in gastrointestinal inflammation and carcinogenesis	Wei Jia	2018	Nature Reviews Gastroenterology & Hepatology	73.082	10.1038/nrgastro.2017.119	556
The gut–liver axis and the intersection with the microbiome	Anupriya Tripathi	2018	Nature Reviews Gastroenterology & Hepatology	73.082	10.1038/s41575-018-0011-z	499
Obesity and cancer risk: Emerging biological mechanisms and perspectives	Konstantinos I Avgerinos	2019	Cell Metabolism	13.934	10.1016/j.metabol.2018.11.001	351
The role of macrophages in nonalcoholic fatty liver disease and nonalcoholic steatohepatitis	Konstantin Kazankov	2019	Nature Reviews Gastroenterology & Hepatology	73.082	10.1038/s41575-018-0082-x	326
Lipotoxicity and the gut-liver axis in NASH pathogenesis	Fabio Marra	2018	Journal of Hepatology	30.083	10.1016/j.jhep.2017.11.014	325
Global epidemiology of NAFLD-related HCC: trends, predictions, risk factors and prevention	Daniel Q Huang	2021	Nature Reviews Gastroenterology & Hepatology	73.082	10.1038/s41575-020-00381-6	299
Probiotics modulated gut microbiota suppresses hepatocellular carcinoma growth in mice	Jun Li	2016	Proceedings of the National Academy of Sciences	12.779	10.1073/pnas.1518189113	286

HCC, Hepatocellular carcinoma; IF, Impact Factor.

### Analysis of keywords

**Figure 9:**
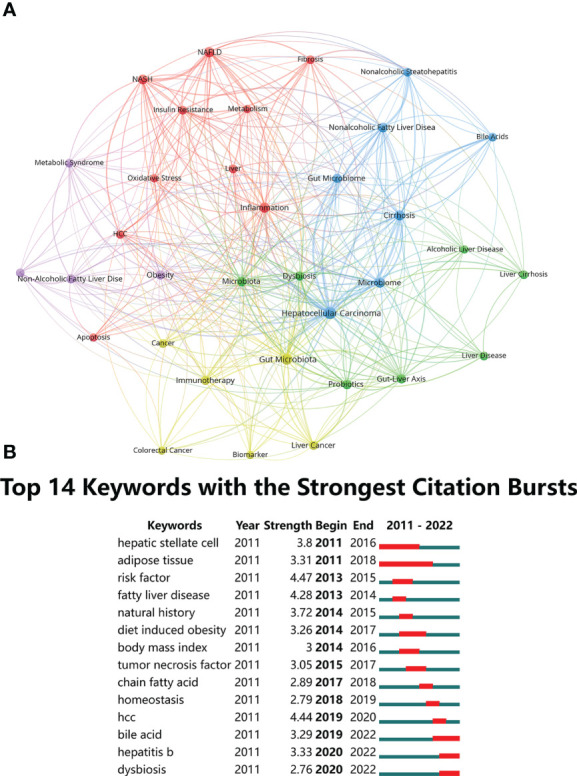
**(A)** The co-occurrence network of keywords. The lines between nodes represent co-occurrence between different keywords. Each node represents one keyword. Circle size is based on the number of occurrences. Different colors represent different items. There are five items in the figure. **(B)** Top 14 Keywords with the strongest citation bursts.

Based on the literature keyword co-occurrence analysis, the detailed description of hotspots topics in the research field were presented. By analyzing the keywords of the 739 publications, a total of 1536 keywords were found, and VOSviewer identified 34 hotspots keywords that appeared at least 15 times and visualized connection with a network map (**Figure 9A**). **Table 8** lists the top 10 hotspots keywords with the highest frequencies. Analysis of keywords with the strongest citation bursts “Burst words” represent keywords that are cited frequently over a period of time, thereby indicating the academic frontier in this field. In present study, the CiteSpace software was used to identified the keywords with strong citation bursts and 14 “Burst words” were presented. **Figure 9B** displays the top 14 “Burst words” as citations in the connection of HCC and gut microbiota during the period of 2011-2022. The red strip indicates the period that the burst keywords maintain. Amongst them, the most keywords with strong citation bursts, risk factor, began in 2013 and end in 2015 with highest strength of 4.47, which was associated with the hepatocarcinogenesis. The second strength of 4.44 was HCC, during 2019-2020 that was associated with the concept of the connection of gut microbiota and HCC. Bile acid, hepatitis B and dysbiosis were the latest keywords with strong citation bursts, indicating that these may be a hot topic in recent years.

**Table 8:** The top 10 hotspots keywords with the highest frequencies.

Rank	keywords	occurrences	total link strength
1	Hepatocellular carcinoma	157	116
2	Gut microbiota	103	68
3	Microbiome	49	49
4	Cirrhosis	48	52
5	Inflammation	47	46
6	NAFLD	46	52
7	Microbiota	44	41
8	Nonalcoholic fatty liver disease	39	34
9	Probiotics	35	30
10	NASH	33	42

## Discussion

Liver cancer ranks the second location among all causes inducing cancer-related death worldwide, and HCC is the commonest malignant tumor of primary liver cancer (1, 2). Hepatocarcinogenesis occurrence has various causes, involving factors including environmental factors, cell cycle metabolism, and the immune system, all of which are associated with activation of proto-oncogenes and inactivation in tumor suppressor genes (16). Hepatitis B virus (HBV) infection, Hepatitis C virus (HCV) infection, alcohol abuse and nonalcoholic fatty liver disease (NAFLD) is currently the most common cause of chronic hepatitis and HCC worldwide (17, 18). Some studies have found that the gut microbiota has significant influence in the development of liver cirrhosis, liver ischemic injury, NAFLD, HCC, and liver graft rejection (19–22). Additionally, the gut microbiotas of HCC patients are significantly different from those of normal subjects, and changes in the composition of gut microbiotas could reveal to grade severity or progression of illness in HCC (23, 24). Therefore, further exploration of gut microbiota promotes an understanding of the tumorigenesis and therapeutic strategies of HCC.

In present bibliometric analysis, we screened 739 publications including 383 articles and 356 reviews on the connection between gut microbiotas and HCC research after searching the WoSCC database. The number of studies related to gut microbiotas and HCC has been steadily increasing since 2011, with approximately ten times as many publications in 2021 as in 2011. The rapid increase in the number of publications over the past decade, indicating that the research of gut microbiota has attracted the attention of HCC research. There have been a number of studies on the involvement of gut microbiota in HCC, including the mechanism of hepatocellular carcinogenesis and tumor drug resistance, diagnostic markers, and treatment strategy (25, 26).

We first analyzed the contributions of countries, institutions, journals and authors in the research of the connection between gut microbiota and HCC. China and the USA are the two countries with the largest number of published papers in this field. Meanwhile, articles published by American scholars have the second average number of citations, only after Spain, which suggest that USA was the most influential country in gut microbiota and HCC research. These were closely related to the fact that the United States has the most advanced scientific research equipment and the largest number of scientific researchers in the world. Although China is far ahead of other countries except the USA in the number of articles published. However, the average citations of articles published by Chinese scholars are in the bottom of the top 10 publications productive countries. This means that Chinese scholars need to concentrate on more meaningful research results, rather than winning over other countries in terms of quantity. The University of California, San Diego in the United States and Shanghai Jiaotong University in China have the highest number of published papers and average citation. there have made tremendous contributions to the study of the gut microbiota in the occurrence and development of liver cancer. Their pioneering research has shed new light on the complex relationship between the microbiome and cancer, deepening our understanding of the mechanisms underlying this deadly disease. Their groundbreaking work represents a true testament to the power of scientific research and the immense potential for collaboration in tackling some of the most pressing health challenges of our time. However, there is very little collaboration between the two research institutions. It is hoped that different research institutions from different countries will have more collaboration in the future. Meanwhile, we found that a large number of publications are primarily distributed in economically developed countries and universities with high academic standing. These factors are closely related to the research resources, research culture, and international collaboration. Economically developed countries and universities with high academic standing have more research resources, invest significant funds to support scientific research, and foster research cultures that encourage innovation and exploration. Additionally, they have more opportunities for international exchange. Moreover, Ohtani N, a renowned scholar from Japan, holds the highest average citation record in his field. His unwavering commitment to uncovering the intricate interplay between the microbiome and liver carcinogenesis has brought to light a previously overlooked area of cancer biology (27, 28). His groundbreaking insights have paved the way for the development of novel strategies aimed at preventing and treating this devastating disease. The scientific community owes a great debt of gratitude to Ohtani N for his outstanding contributions to the field of cancer research.

**Figure 10:**
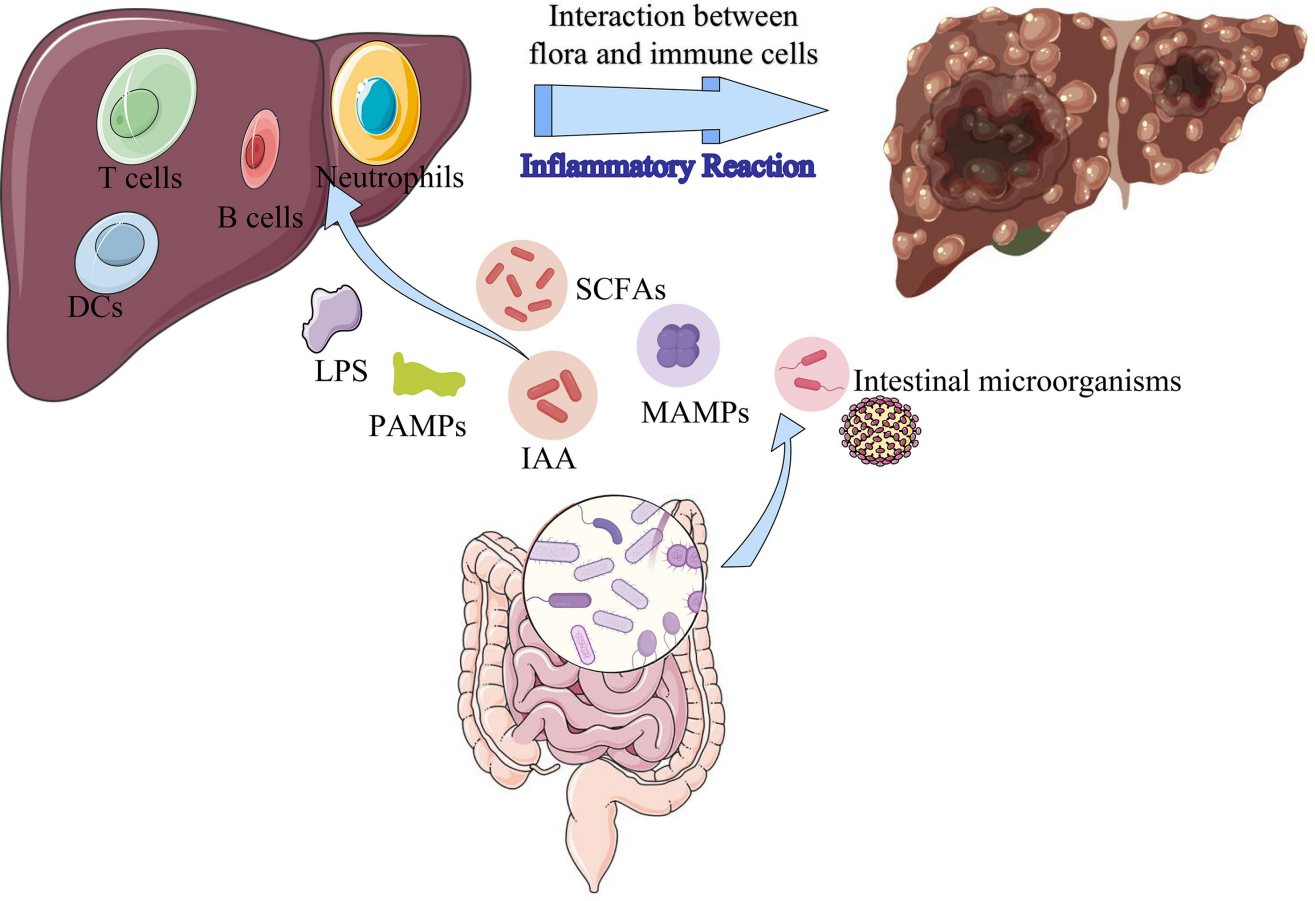
The mechanism of intestinal microbes and its metabolites influencing the formation of HCC. LPS, lipopolysaccharides; SCFAs, short-chain fatty acids; IAA, indoleacetic acid; MAMPs, microbe-associated molecular patterns; PAMPs, pathogen-associated molecular patterns.

Increasing evidence has illustrated the association between gut microbiota and tumorigenesis, which is important for exploring the pathogenesis of hepatocarcinogenesis (29, 30). The microbiota-gut-liver axis is a physiological and anatomical communication system that integrates intestinal microbiota and immunological signals with gastrointestinal tract and liver (31, 32). Recent studies have demonstrated the stability of the gut microbiota and its metabolites is important factor for the maintenance of normal liver functions (33, 34). Exposure of the chronic hepatitis to the dysbiosis of the gut microbiota and their derived metabolites results in cirrhosis and ultimately to hepatocarcinogenesis (32, 35, 36). Various intestinal microorganisms, intestinal microbial-derived metabolites, and cellular components (peptidoglycans, flagellin, lipoteichoic acid, and lipopolysaccharide), intestinal epithelial cell exosomes are transported into the liver *via* the portal vein or biliary system and interact with intrahepatic immune cells, causing inflammatory immune response and inducing normal liver from pre-cirrhotic stages to cirrhosis, liver cancer (37) (**Figure 10**). LPS activates the TLR4 receptor of HCC cells through NF- κ B pathway induces epithelial mesenchymal transition (EMT), which is an important molecular mechanism of tumor cell proliferation and metastasis (38). Short-chain fatty acids (SCFAs) are generated in the colon by the fermentation of dietary fiber by gut microbiota. Hepatocytes can utilize SCFAs as a source of energy, which can lower glucose production by the liver and enhance insulin sensitivity (39, 40). SCFAs can also stimulate the secretion of intestinal hormones, including glucagon-like peptide-1 (GLP-1), which can further enhance insulin secretion and improve glucose metabolism (41). Indoleacetic acid (IAA) is a plant hormone that can be produced by certain bacteria in the gut microbiota. IAA exerts its effects by binding to specific receptors on the surface of bacterial cells, which can activate or inhibit various signaling pathways that control gene expression and metabolism (42). Additionally, IAA can stimulate the production of certain enzymes that break down complex carbohydrates, making them more accessible to other microbes (42). It can also influence the production of other hormones and signaling molecules that affect the growth and activity of other bacterial species (43). When the intestinal flora is damaged, the intestinal mucosal barrier and immune functions is destroyed, leading to intestinal endotoxemia (IETM), bacterial translocation, and excessive absorption of bacterial metabolites (44, 45). These factors contribute to the process of liver cell inflammation, damage, and repair, especially on the basis of the original liver disease, HCC can develop due to hepatocyte damage and repair during inflammation.

There are also many studies on gut microbiota in the treatment of HCC (46, 47). Fessas P et al. found that in the mouse tumor model, the use of antibiotics will lead to the disorder of intestinal flora, which will cause changes in the composition of immune cells in the liver, thus affecting the growth of tumors in the liver (48). Probiotics are living microbes that confer beneficial effects to the host and help to maintain the stability of an organism as a whole. Probiotics can reduce toxic chemical induced hepatocarcinogenesis by rebuilding intestinal homeostasis and improving intestinal and liver inflammation (49). Research shows that dysbiosis of intestinal microbiota may be one cause of HCC, and probiotics that restore intestinal microbiota function are an effective and safe strategy to treat HCC (50, 51). Fecal microbiota transplantation (FMT) is the transplantation of fecal microbiota suspension from a healthy pre-screened donor to an individual with disease (52). Some studies have also found that FMT may effectively improve the immunotherapeutic response in HCC (53–55).

With the rapid development of high-throughput sequencing technologies such as single-cell sequencing, 16s RNA sequencing, genomics and spatial transcriptomics, numerous studies have been conducted to investigate the microbial diversity and biological functions of intestinal microbes in hepatocarcinogenesis. The crucial next step in exploring the influence of gut microbiota on the occurrence and progression of HCC is to integrate microbiome data, experimental results, and clinical data. By concentrating on the pathogenesis of HCC, we will search for new key targets that influence the development of HCC between the liver and the intestine. Meanwhile, future studies need to further explore the relationship between gut microbiota and HCC occurrence, development, and treatment and explore new strategies for regulating gut microbiota.

This study is the first to use bibliometric method to explore the connection between gut microbiotas and HCC research in the past decade. There are several limitations to this study that are worth noting. First, our publications were derived entirely from the WOSCC database and do not include publications of other databases, such as Google Scholar, PubMed and MEDLINE. Second, our research is mainly based on English-language literature, so we may be lost some high-quality literature published in other languages. We hope that those limitations can be elaborated in the future.

## Conclusion

In present study, we explored the links between gut microbiota and HCC field with the method of bibliometric methods. The connection between gut microbiota and HCC field have always been enthusiastically investigated by researchers. Through bibliometric analysis, we found that gut microbiota is crucial in the pathogenesis and oncotherapy of HCC through published literature review.

## Data availability statement

The original contributions presented in the study are included in the article/supplementary material. Further inquiries can be directed to the corresponding authors.

## Author contributions

ZC and CD downloaded and analyzed the data. ZC, YG, QL designed the study. ZC, CD, and YH drafted the manuscript. QL, SZ, and YG reviewed and revised the manuscript. ZC and BC edited the figures and tables of the article. All authors contributed to the article and approved the submitted version. All authors met the ICMJE authorship criteria.
